# High‐Tech Augmentative and Alternative Communication in a Burn Patient With Voicelessness: A Case Study

**DOI:** 10.1111/nicc.70496

**Published:** 2026-04-23

**Authors:** Silvia Porcarelli, Monica Codara, Diletta Fabrizi, Michela Luciani, Davide Ausili

**Affiliations:** ^1^ ASST Grande Ospedale Metropolitano Niguarda Milan Italy; ^2^ Nursing Sciences and Public Health, University of Rome Tor Vergata Rome Italy; ^3^ Fondazione IRCCS San Gerardo dei Tintori Monza Italy; ^4^ School of Medicine and Surgery, University of Milano–Bicocca Monza Italy

**Keywords:** burn, communication devices for people with disabilities, intensive care unit, nursing, patient satisfaction, technology

## Abstract

Loss of speech due to mechanical ventilation is common among patients admitted to intensive care. This condition, often referred to as voicelessness, can generate negative emotions such as frustration and anger. Augmentative and Alternative Communication (AAC) techniques are useful for facilitating communication, but their use can be complex in patients with extensive burns. This case study describes the use of a high‐tech AAC system based on a motion sensor in an adolescent with second‐ and third‐degree burns covering 60% of the total body surface area, requiring both invasive and non‐invasive mechanical ventilation. Due to injuries to their hands and neuromuscular weakness, the patient was unable to use conventional low‐ or high‐tech AAC devices. The use of the motion‐sensor‐based AAC system enabled the patient to communicate specific needs and formulate complex sentences, facilitating individualised nursing care. The patient and family reported high satisfaction with the quality of care, while nurses noted a positive impact on the care relationship without increasing their workload. This case highlights the importance of personalised communication strategies in critically ill burn patients and suggests that the adoption of appropriate AAC systems may reduce missed nursing care and improve emotional and care outcomes.

1

**Impact Statement**

- What is already known?
  ○ Voicelessness due to mechanical ventilation is a frequent and distressing experience for patients in the Intensive Care Unit, often leading to significant psychological frustration and anxiety.
  ○ Effective communication, supported by Augmentative and Alternative Communication (AAC), is essential for patient safety, as interaction barriers are linked to unmet clinical needs and a higher risk of missed nursing care.
  ○ There is a significant lack of clinical evidence and practical guidelines regarding the implementation of AAC for patients with extensive burns, where standard communication tools are often unusable.
- What this paper adds?
  ○ This case study identifies specific physical obstacles, such as hand dressings and extreme neuromuscular weakness, that can make conventional AAC methods hard to implement in the context of severe burns.
  ○ It illustrates the feasibility and application of a motion‐sensor‐based high‐technology AAC system for an adolescent with burns covering 60% of the total body surface area, enabling a transition from communicating basic needs to complex sentences.
  ○ The findings suggest that tailoring communication technology to the unique physical limitations of burn patients has the potential to enhance the therapeutic relationship and family satisfaction, highlighting the importance of personalized interventions in specialized critical care.

## 2 Introduction

The inability to speak due to mechanical ventilation, or voicelessness, affects from 30% to 100% of patients in Intensive Care Units (ICU) [1]. Patients' inability to express needs and thoughts leads to negative emotions, such as anger, fear and stress [2, 3] and it is believed to play a role in the development of Post Intensive Care Syndrome [4]. Nurses have been reporting consistently that voicelessness affects the relationship with patients, leading to frustrations on both sides [5] and causing missed care, unmet needs and overtreatment [6].

Alternative and Augmentative Communication (AAC) strategies have been used to facilitate communication in voiceless patients [7]. AAC encompasses techniques to enhance or substitute speech [1] divided into low‐tech (e.g., pen and paper, alphabet boards, lip reading) and high‐tech (e.g., eye tracking devices, apps and tablets). Since there is no evidence that any single technique is superior, the current consensus supports the use of personalised approaches based on patients' needs, residual capacities and clinical conditions [8]. The use of AAC strategies has an impact on improving communication efficacy and health outcomes, such as reduced anxiety, agitation and increased quality of life [1, 9].

Currently, no studies have investigated the use of AAC in patients with burn injuries. The clinical presentation of these patients might hinder the use of AAC, both high‐tech and low‐tech. This case study therefore illustrates the use of a high‐tech AAC in a patient with burn injuries.

## 3 Methodology

The current case study has been reported according to CARE guidelines [10].

## 4 Case Presentation

The patient is a 16‐year‐old adolescent admitted to the ICU of a tertiary hospital in northern Italy with a diagnosis of second‐ and third‐degree burn injuries covering 60% of the total body surface area [11] and lung damage caused by inhalation of combustion fumes. The burns covered the head, face, upper limbs, hands, part of the chest, the entire back and parts of the lower limbs. The patient was transferred from another hospital in which they received care for the first 12 days after the incident. During that period, the patient required orotracheal intubation and invasive mechanical ventilation with deep sedation through multidrug regimen sedatives, anaesthetics, neuromuscular blockers, due to the severity of the inhalation injury and the need for multiple escharotomy procedures and skin grafting. During this period, the patient maintained a Richmond Agitation‐Sedation Scale (RASS) score [12] between −4 and −5, indicating deep sedation to an unarousable state. For this reason, it was not possible to establish any form of communication between the patient and their family members or the healthcare team. For the first 3 days of hospitalisation in the current ICU, the patient's sedation and analgesia were pharmacologically maintained through the administration of appropriate doses of propofol, midazolam, remifentanil, dexmedetomidine and ketamine, as well as intravenous delorazepam and enteral promazine. The aim was to provide a less profound level of sedation, equivalent to −1/0 on the RASS Scale, and to ensure the absence of pain. On the fourth day of hospitalisation, the patient underwent surgery with autologous graft harvesting and coverage, requiring deep sedation and curarisation.

### 4.1 The Need to Communicate

During the patient's hospitalisation in the ICU, the nurse‐to‐patient ratio was 1:1. The nurse assessed the patient's need to communicate and intervened to facilitate communication through available AAC strategies. The patient gradually showed the need to communicate through upper limb movements, eye contact and nodding (yes/no) in response to questions from healthcare staff. Due to the dressings on their face, it was not possible to interpret lip movements. It was not possible to use the low‐tech AAC tools available in the ward (pen and paper, alphabet/icons board) or the patient's personal mobile phone because, due to burns to their hands, the patient would not have been able to perform fine movements such as writing, pointing to letters on the alphabet board or using a touch screen. Despite the burns to the patient's face, they never showed any hearing loss or visual impairment (with or without glasses). The eye examination did not reveal any damage that could alter their vision. The patient never showed any cerebral or cognitive deficits or signs of delirium during their hospitalisation. The patient's body movements were partially restricted by dressings covering 60% of his body and their positioning on the fluidised bed.

### 4.2 Intervention

Following a thorough nursing assessment, which included an evaluation of the patient's level of sedation/agitation, the absence of delirium (level of alertness, level of consciousness and organised thinking) and the patient's communication abilities, as well as an assessment of their mobility, AAC was identified as the only suitable approach and was proposed to the patient, who accepted it. Furthermore, dichotomous questions with yes/no answers using head nods are closely linked to the operator's questions, especially if, as in this case, it was not initially possible to read lips due to the dressings. Moreover, it was not possible to investigate the patient's specific needs, which were considered relevant for personalising nursing care. The rationale behind the choice of this high‐tech communication method was explained to the patient. This specific AAC system is a software running on a tablet that uses a motion sensor. The system supports three operating modes. In the single‐gesture mode, icons and letter cells were automatically highlighted in sequence at a rate set by the device, and the user performed a single gesture to select the desired target. In the two‐gesture mode, one gesture was used to move the highlight across icons or letter cells, and a second gesture was used to select the highlighted target. Finally, a manual mode allowed direct interaction via the touchscreen. Touchscreen input could also be used in combination with the motion‐sensor modes. The sensor can be positioned on different body parts (e.g., head, hands/arms, feet) and requires a minimal movement so that even those with severely impaired mobility can utilise it. The device can be used either in an icon‐based mode showing on a hierarchical nested model several needs or emotions (semeiotic table) or on a simplified keyboard (word prediction function).

This high‐tech AAC was available in the ICU thanks to a partnership between the hospital, the University, and a technology company, developed within the framework of a nursing study. Its use for this patient was outside the scope of the study and was based exclusively on the principles of Good Clinical Practice and professional ethics. The nurses who cared for the patient completed a 2‐h training course on the use of the device together with the company's specialist and a University researcher. The patient was provided an overview of how to use the device and was instructed to select a body part capable of performing a repeatable movement, where the motion sensor would be placed. The patient selected the right arm (dominant arm) as the location for the sensor and chose an up–down movement of the forearm that was compatible with the dressings and comfortable to perform. The single‐gesture mode was selected. The device analysed the motion sensor signal while the arm was at rest and during the selected movement to enable calibration. The nurse then demonstrated how the movement could be used to select icons on the semiotic table and letters on the keyboard. Training lasted approximately 15–20 min, during which the patient learned to use the semeiotic table and tested the reliability of the movement detected by the sensor. While using the device, the patient mainly used the semeiotic table and only in some cases used the keyboard to write complex sentences. The patient's parents received a detailed explanation of the device as soon as they arrived for their visit, and its use was immediate and intuitive without the need for repeated training. The patient and their parents were assisted throughout its use by the nurses. The device was used for 48 h until the scheduled extubation and for an additional 24–32 h during non‐invasive mechanical ventilation with an orofacial mask, at the patient's request.

## 5 Results

**FIGURE 1:**
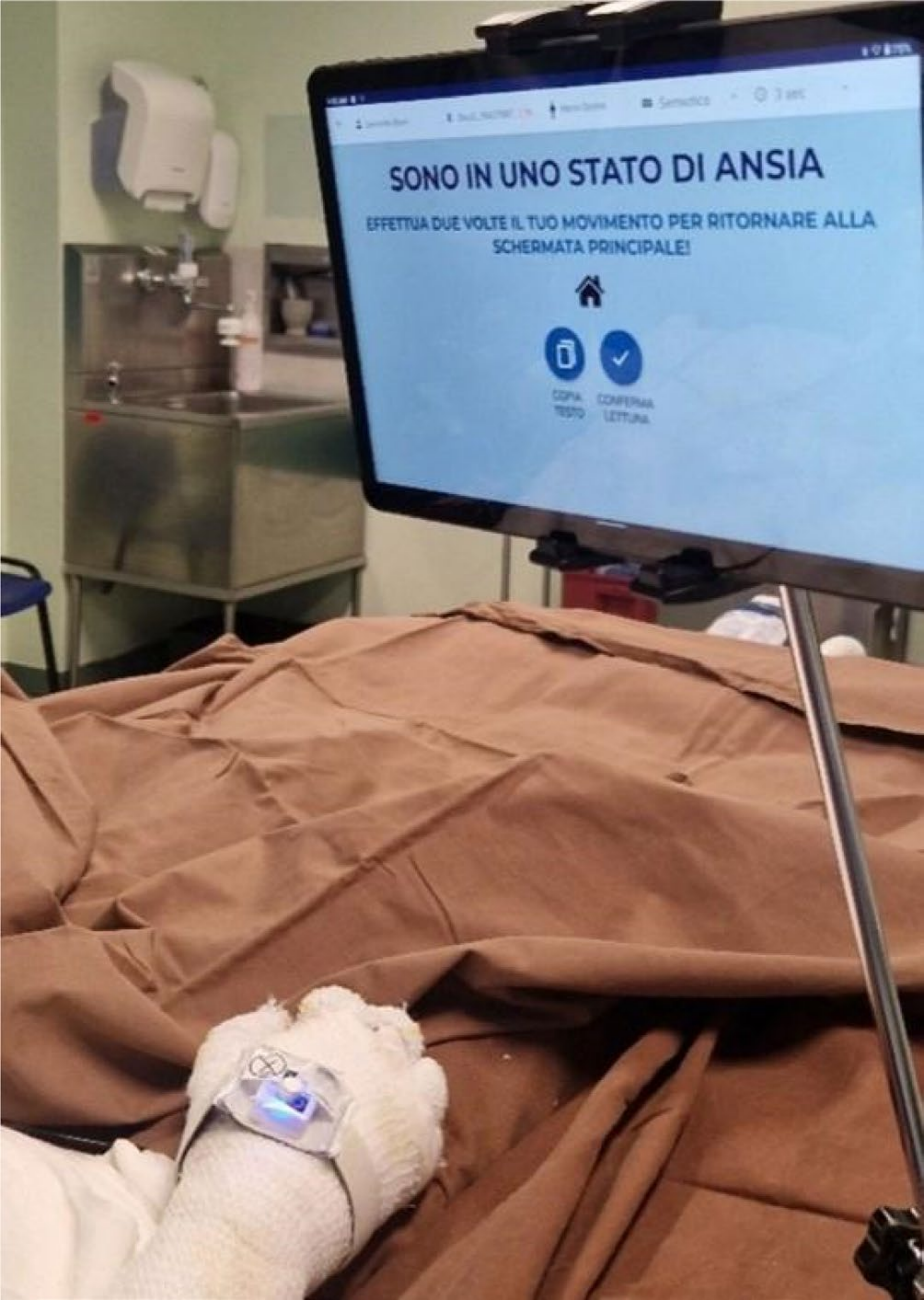
The patient with the device in use with the sensor on the right wrist and the tablet showing the emotion chosen by the patient on the semiotic table (‘I am in a state of anxiety’).

Using the chosen AAC (Figure 1), the patient was able to communicate specific sensations, emotions and needs (Table 1).

**TABLE 1:** The patient's emotions, symptoms, nursing care needs, needs for interaction in communication (semeiotic table) and written communications (keyboard mode).

*Emotions* (semeiotic table)
I am feeling very angry.
I am in a state of anxiety.
I am feeling a bit lonely.
I am feeling scared.
I am feeling sad.
I am feeling better today.
I am experiencing happiness.
*Symptoms* (semeiotic table)
My right eye is itchy.
My left eye is itching.
I am feeling nauseous.
My head is itchy.
I have grade X chest pain.
I have grade X pain in my neck.
I have grade X pain in my back.
I have grade X pain when breathing.
I have grade X pain in my stomach.
I have grade X pain in my right shoulder.
I have grade X pain in my right elbow.
I have grade X pain in my right hand.
I have grade X pain in my left hand.
*Nursing care needs* (semeiotic table)
Need for interaction in communication
I need someone immediately.
When will I be able to return home?
What day and time is it?
I would like to use the keyboard.
I would like to know when the tube will be removed.
When will I be able to see my family?
I would like to know how my recovery is progressing.
I would like to speak to a nurse.
I would like to speak to a doctor.
I would like to communicate with my father.
I would like to communicate with my mother.
Need to breathe
I have trouble breathing. I am short of breath.
I have a lot of mucus today.
Need to eat and hydrate
I am very thirsty.
I am very hungry.
Urinary and intestinal elimination needs
I need to urinate.
I need to defecate.
Hygiene needs
I would like to arrange my sheets.
I need to wash myself.
I need to brush my teeth
Need for movement
I would like to adjust my pillow.
I would like to lower the angle of my bed.
I would like to turn over in bed on my right side.
I would like to increase the inclination of my bed.
Need for rest and sleep
I would like to sleep now.
Need to maintain cardiovascular function
I am very cold.
I am very hot.
*Communications typed on the digital keyboard* (keyboard mode)
51 [written to indicate the television channel]
Nurse, water, he did it. [Pointing to the drinking straw]
Some apps that used to be there are missing.
Good night.
Restore my screen to how it was before.
Once the tube has been removed, will I be able to drink?
Mum, can you bring me some food from home?
I need to tell mum to bring rice and coconut milk, an orange Crodino, the bars and the water bottle.
Did you tell them? [Referring to the question above]

This facilitated the timely recognition of patient needs and the ability to accurately personalise nursing care. Furthermore, the patient was able to express complex sentences using the keyboard, such as specific requests to their parents or nursing staff (Table 1). The ACC remained available to the patient throughout the day. During the night, the monitor was switched off to favour sleep, and it was available at the patient request. The patient preferred to use the device also during non‐invasive mechanical ventilation, as it allowed them to communicate with the full‐face mask without interfering with the ventilator.

### 5.1 Patient and Family Outcomes

The patient reported an improvement in the perceived quality of care, as the ability to communicate specific needs to the nurse enhanced their sense of comfort and safety. Both parents reported feeling ‘indescribable happiness’ at being able to communicate with their child and learn about their feelings and thoughts.

### 5.2 Healthcare Personnel Outcomes

Four out of five nurses who assisted the patient during the use of high‐tech AAC reported an improvement in the perceived quality of care provided, while one nurse reported no perceived difference, compared with the period prior to the use of the device. Three out of five nurses reported that high‐tech AAC facilitated and enhanced the care relationship, enabling them to respond to very specific needs that they would otherwise have been unable to recognise (e.g., itchy left eye). No nurses reported an increase in workload. The medical staff (five out of six) expressed interest and excitement about the patient's use of the device.

## 6 Discussion

The analysis of this clinical case highlights, in accordance with the literature, how the choice of communication method must be tailored to the patient's needs and abilities [8]. Due to the severity of the burns to their hands and neuromuscular weakness caused by immobilisation and drug treatment, the patient was unable to use low‐tech AACs or most readily available high‐tech AACs, for example, personal devices. The effectiveness of communication with the patient seems to suggest a decrease in distress and anger related to the inability to communicate, in line with existing literature [2, 3]. Although these effects do not directly alleviate anxiety and fear related to hospitalisation and clinical conditions, the use of AAC allows the patient to express their feelings and needs, enabling staff to implement multidisciplinary mitigation strategies.

Looking at the communication content, it was possible to implement corrective measures for the patient's pain and comfort based on their feedback. Unlike closed‐ended questions (yes/no) or lip reading, which often limit the information patients can provide, high‐tech AACs can facilitate the recognition of very specific physical and emotional needs. This strategy reduces frustration for both patients and nurses [5, 13] and allows unnecessary interventions to be reduced while meeting the patients' needs [6]. Furthermore, this specific AAC requires a limited training time compared with, for example, eye‐tracking devices. Training can be further shortened for patients who are digitally literate, as is generally the case for adolescents and young adults.

The literature highlights that, in ICUs, nursing activities addressing patients' physiological and emotional needs are the most frequently neglected [6, 14]. In an ICU setting where care priorities are based on clinical criticality, patients' communication needs are often overlooked. Burn patients are critical patients who, on average, face long periods of intensive care [15], often without the ability to speak and ignoring this need can lead to negative outcomes such as emotional distress [4] and the development of Post Intensive Care Syndrome. The implementation of various communication strategies could reduce this type of missed nursing care and positively affect both short‐ and long‐term outcomes.

## 7 Patient Perspective

The patient reported that being able to communicate after such a long time was a meaningful and highly reassuring experience. Promptly notifying staff of their needs contributed to a greater sense of safety and reduced feelings of loneliness. Furthermore, compared to previous attempts at communication using eye contact and/or nodding (yes/no), the patient felt that their needs were better understood. They also reported feeling much more comfortable using the device to communicate with their family. Overall, despite the challenges of intubation and intensive care, the patient felt that using the AAC device helped them cope and enhanced their sense of well‐being.

## 8 Implications for Future Research

There are heterogeneous studies in the literature on the use of AACs in voiceless patients, both in terms of study design and outcome measurement [4, 16, 17]. This clinical case suggests the need for primary studies investigating the use of AAC strategies in voiceless patients undergoing invasive and non‐invasive mechanical ventilation in ICUs. Further studies should address this gap for improving care for adult and paediatric burn patients.

## 9 Conclusion

The results of this case study highlight the importance of using AACs with a personalised approach. These tools could allow the patients' specific needs to be addressed without increasing workload, while also improving the perceived quality of care for the patient, their family and the nursing staff.

## Author Contributions

**Silvia Porcarelli:** conceptualization, methodology, formal analysis, investigation, resources, data curation, writing – original draft preparation and visualisation. **Monica Codara:** conceptualization, resources, data curation, writing – original draft preparation, writing – review and editing and project administration. **Diletta Fabrizi:** conceptualization, methodology, formal analysis, investigation, resources, data curation, writing – review and editing, supervision and project administration. **Michela Luciani:** conceptualization, methodology, investigation, resources, writing – original draft preparation, writing – review and editing, supervision and project administration. **Davide Ausili:** conceptualization, methodology, investigation, writing – review and editing, supervision, project administration and funding acquisition. All authors have read and agreed to the published version of the manuscript.

## Funding

The authors have nothing to report.

## Ethics Statement

This case study was submitted and approved by the Ethics Committee ‘Comitato Etico Territoriale Lombardia 3’ (approval ID: 6917_11.02.2026_N). Written informed consent and privacy forms were obtained from the patient (minor) and their parents, following the COPE guidelines [18].

## Conflicts of Interest

The authors declare no conflicts of interest.

## Data Availability

The data that support the findings of this study are available on request from the corresponding author. The data are not publicly available due to privacy or ethical restrictions.
